# Patient Specific Dosimetry Phantoms Using Multichannel LDDMM of the Whole Body

**DOI:** 10.1155/2011/481064

**Published:** 2011-09-25

**Authors:** Daniel J. Tward, Can Ceritoglu, Anthony Kolasny, Gregory M. Sturgeon, W. Paul Segars, Michael I. Miller, J. Tilak Ratnanather

**Affiliations:** ^1^The Center for Imaging Science, The Johns Hopkins University, Baltimore, MD 21218-2686, USA; ^2^Carl E. Ravin Advanced Imaging Laboratories, Duke University, Durham, NC 27705, USA; ^3^Department of Biomedical Engineering, University of North Carolina, Chapel Hill, NC 27599-7575, USA; ^4^Department of Radiology, Duke University Medical Center, Durham, NC 27710, USA; ^5^Institute for Computational Medicine, The Johns Hopkins University, Baltimore, MD 21218-2686, USA

## Abstract

This paper describes an automated procedure for creating detailed patient-specific pediatric dosimetry phantoms from a small set of segmented organs in a child's CT scan. The algorithm involves full body mappings from adult template to pediatric images using multichannel large deformation diffeomorphic metric mapping (MC-LDDMM). The parallel implementation and performance of MC-LDDMM for this application is studied here for a sample of 4 pediatric patients, and from 1 to 24 processors. 93.84% of computation time is parallelized, and the efficiency of parallelization remains high until more than 8 processors are used. The performance of the algorithm was validated on a set of 24 male and 18 female pediatric patients. It was found to be accurate typically to within 1-2 voxels (2–4 mm) and robust across this large and variable data set.

## 1. Introduction

Measuring the radiation dose a patient accumulates through life is an important matter that has been receiving much attention recently, in particular for growing children (e.g., in the New England Journal of Medicine's recent critique of CT use [1], and the adoption of the Image Gently program [2] by the Society of Pediatric Radiology, the American Society of Radiologic Technologists, the American College of Radiology, the American Association of Physicists in Medicine, and others). While directly measuring dose to individual organs is impractical, the development of computational phantoms containing dosimetric information (e.g., [3]), such as the extended cardiac-torso (XCAT) phantom used in this study [4] have begun to be a reliable substitute. A key shortcoming of this strategy is that standard phantoms cannot adequately reflect variability between patients, especially for children of different sizes and ages, and defining new phantoms for each patient manually would be unfeasible. The strategy used here consists of manually segmenting a small subset of organs from pediatric CT data and calculating a full body mapping to a similarly segmented adult XCAT phantom [5]. The resulting transformation is used to map rich anatomical and dosimetric information to the child's body.

To map dense image data as well as point-based manifold data between adult and child, this application requires a smooth invertible transformation (a diffeomorphism) to be defined everywhere on the background space of the CT scan. Such transformations are an important focus of computational anatomy [6], where anatomical variability is understood by studying diffeomorphisms mapping anatomical manifolds to one another. Formally, anatomy is modelled as the quadruple (*Ω*, *𝒢*, *ℐ*, *𝒫*), where *Ω* is the background space (i.e., subsets of ℝ^3^), *𝒢* is a group of diffeomorphisms on *Ω*, *ℐ* is the orbit of a template *I*_0_ under *𝒢*, and *𝒫* is a family of probability measures on *𝒢*. Geodesic paths, *ϕ*_*t*_ ∈ *𝒢* for *t* ∈ [0,1], are used to evolve a template according to *I*_0_∘*ϕ*_*t*_^−1^, and a mapping to a target *I*_1_ is defined when *I*_1_ = *I*_0_∘*ϕ*_1_^−1^.

Large deformation diffeomorphic metric mapping (LDDMM) [7] generates such mappings (*ϕ*_1_(*x*)) by integrating a smooth time dependent velocity field *v*_*t*_(*x*) [8],

(1) $$\phi_{t}\left(x\right)=\overset{t}{\underset{0}{\int }}v_{t^{\prime }}\left(\phi_{t^{\prime }}\left(x\right)\right)dt^{\prime },$$

with the initial condition being identity, *ϕ*_0_(*x*) = *x*. A functional of the velocity field, which enforces image matching as well as smoothness and ensures the path is a geodesic, is minimized as discussed below.

## 2. The Multichannel LDDMM Algorithm

There are existing algorithms for full body image registration, which are used (e.g.) in registering PET to CT data [9–11] and compensating for deformations such as breath holds. However, these tend to use elastic models, which are suitable for describing the small deformations that register two images of the same patient but are unable to accurately describe the widely varying deformations between adults and children of various ages. In addition to the constraints on smoothness and invertibility, transformations generated by LDDMM are well suited to this application, because its fluid model (rather than elastic) allows for large deformations to be generated [12] and because the submanifold preserving property of diffeomorphisms [13] allows a transformation calculated from a handful of segmented structures to be accurately applied to the thousands of anatomical structures defined in the XCAT phantom. Moreover, additional properties are well suited to future exploration. For example, LDDMM allows metric distances to be defined between template and target anatomies [8, 14] and allows statistical codification of anatomy [15, 16].

In this work, we use multichannel LDDMM (MC-LDDMM), an algorithm which treats each segmented organ as a separate image linked by a common background space [17] to calculate diffeomorphisms. This is accomplished by calculating the velocity field minimizing the energy functional

(2) $$E=\int dt{||Lv_{t}||}_{2}^{2}+\overset{M}{\underset{i=1}{\sum }}\frac{1}{\sigma_{i}^{2}}{||I_{0}^{i}\circ \phi_{t=1}^{-1}-I_{1}^{i}||}_{2}^{2},$$

where *I*_1_^*i*^ and *I*_0_^*i*^ are the *i*th (out of *M*) channels (organs) of the target and template images, *ϕ*_*t*=1_ is a diffeomorphism generated by integrating the velocity field *v*_*t*_ from *t* = 0 to 1, and *σ*_*i*_^2^ describes the contribution of the *i*th channel to the overall energy. The operator *L* = −*γId* + *α*∇^2^, where *γ* = −1 is fixed and *α* is varied, *Id* is identity, and ∇^2^ is the Laplacian operator, ensures smoothness of the velocity field and resulting deformations, with larger *α* corresponding to smoother deformations, and smaller *α* corresponding to more accurate transformations.

The energy gradient can be computed as [17]

(3) $$\nabla_{v}E_{t}=2v_{t}-K\left[\overset{M}{\underset{i=1}{\sum }}\frac{2}{\sigma_{i}^{2}}\left|D\phi_{t,1}\right|\nabla J_{t}^{0i}\left(J_{t}^{0i}-J_{t}^{1i}\right)\right],$$

where *K* is the operator inverse of *L*^†^*L*, |·| denotes determinant and *D* denotes the Jacobian. The transformation generated by integrating (1) from time *t*′ = *s* to time *t*′ = *t* is denoted *ϕ*_*s*,*t*_ (i.e., *ϕ*_*s*,*t*_ = *ϕ*_*t*_∘*ϕ*_*s*_^−1^ = *ϕ*_*t*,*s*_^−1^). The quantity *J*_*t*_^0*i*^ is the *i*th template channel transformed up to time *t* (i.e., *J*_*t*_^0*i*^ = *I*_0_^*i*^∘*ϕ*_*t*_^−1^ = *I*_0_^*i*^∘*ϕ*_*t*,0_), *J*_*t*_^1*i*^ is the *i*th target channel transformed backwards from time 1 to time *t* (i.e., *J*_*t*_^1*i*^ = *I*_1_^*i*^∘*ϕ*_*t*,1_), and ∇ is simply the spatial gradient.

It can be seen that the transformation and its inverse must be defined at all times, which was discretized here into 11 equally spaced time points from *t* = 0 to *t* = 1. Calculating this transformation from the velocity field is a large part of the computational load. Integration in time is performed using semi-Lagrangian advection, a technique used in numerical weather prediction [18]. We use an implicit method for numerical integration, with 3 iterations per voxel at each timestep.

Moreover, a deformed target and template image must be calculated at each timestep. We use trilinear interpolation, which corresponds to another large computational load. To optimize calculations, the images for each channel were computed in the same loop (loop fusion).

Finally, application of the operator *K* is implemented by multiplication in the Fourier domain. The FFT calculations were performed and parallelized using Intel Math Kernel Library's (MKL) FFT routines.

Since many steps of this algorithm involve independent calculations on a regular 3D voxel grid, it is well suited to parallelization. In our C++ implementation of the LDDMM algorithm, OpenMP (open multiprocessing) library routines were used. As stated in [19], “the OpenMP Application Program Interface (API) supports multi-platform shared-memory parallel programming in C/C++ and Fortran on all architectures. …OpenMP is a portable, scalable model that gives shared-memory parallel programmers a simple and flexible interface for developing parallel applications for platforms ranging from the desktop to the supercomputer.” In our algorithm, at each iteration of gradient descent, different operations defined on data over the voxel grid were parallelized using work-sharing constructs, and loop iterations were split among the threads. The program was compiled using Intel C++ compiler version 12.0, with automatic compiler optimizations. It was run on a Dell R900, a 4 socket node with 6 cores per socket, with an Intel Xeon CPU E7450 at 2.40 GHz.

## 3. Methods

### 3.1. Calculation of Full Body Maps

**Figure 1:**
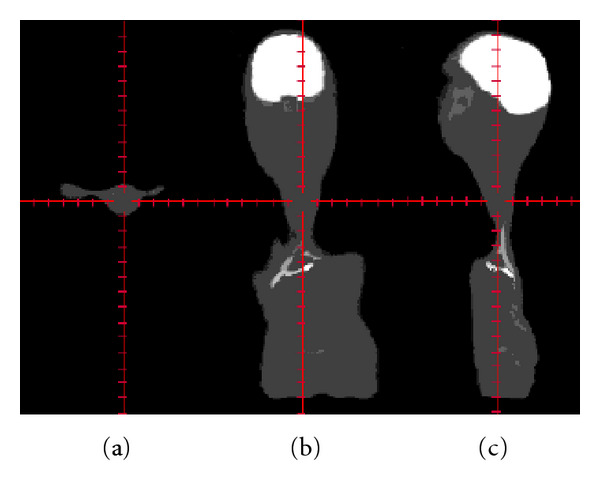
An example of how the standard MC-LDDMM algorithm fails for full body mapping. (a) axial, (b) coronal, and (c) sagittal images of a deformed adult template. Notice that the abdominal organs have been catastrophically shrunk causing distortions in nearby neck and thoracic structures and that details in the face and skull have been lost.

 In previous work [5], the feasibility of using multi-MC-LDDMM for this purpose was explored. A mapping to a single pediatric patient was calculated, and a reasonable subset of segmented organs was determined. However, generalizing this algorithm to a population of patients proved difficult. For example, where initial overlap of organs or bony details between template and target was poor, the diffeomorphism tended to shrink organs close to a point. Such distortions would also negatively affect the registration of nearby structures. Furthermore, when structures were shrunk by the diffeomorphism details were lost, and when structures were expanded, their initial voxelized character was spuriously reproduced at the larger scale. These difficulties are illustrated by showing a deformed adult template in Figure 1, where abdominal organs are seen contracting to a very small size, nearby structures in the neck and thorax are distorted, and features in the face and skull are lost. Further investigation resulted in the algorithm being made more robust [20] but at the expense of increased computation time. 

In the modified MC-LDDMM algorithm, (2) is minimized by initializing the velocity field to 0 and using a gradient descent routine with a large value of *α*. At convergence, the value of *α* is decreased, and minimization resumes, starting with the previously calculated velocity field. This procedure is iterated a total of four times. This sequential reduction of the parameter *α* (denoted “cascading *α*”) allows for a coarse to fine registration and is responsible for the increased robustness as well as increased computation time of the modified algorithm. Beginning with a large value of *α* is analogous to Tikhonov regularization, encouraging a desirable solution to an ill posed problem. The final small value for *α* is chosen to give the desired level of accuracy in our mapping. Decreasing the value for *α* abruptly often results in nondiffeomorphic transformations due to numerical instability. So, we include 2 intermediate values to mitigate this effect and unfortunately must bear the price of considerably increasing computation time.

The MC-LDDMM algorithm with cascading *α* was used to generate mappings between one of two adult templates (one male and one female), and pediatric patients (24 male and 18 female). Each was defined on an 256 × 256 × 520 2mm^3^ voxel grid. The patients varied in size between 0.072 and 0.472 times the volume of the adult, with an average of 0.233 times. Males ranged from 0.072 to 0.472 times the adult volume with a mean of 0.246, while females ranged from 0.076 to 0.372 times the adult volume with a mean of 0.215. The images were segmented into 8 channels with corresponding organs and weightings defined in Table 1, and 87 landmarks were placed automatically [4] mainly on easily reproducible bony structures. Images were initially aligned with an affine transformation minimizing distances between corresponding landmarks, followed by nonlinear landmark LDDMM [21]. Following this, cascading *α* MC-LDDMM was used with the four values *α* = 0.05,0.025,0.01,0.005. In previous work, we found this particular sequence to give qualitatively good results in 2D simulations and 3D full body data [20].

**Figure 2:**
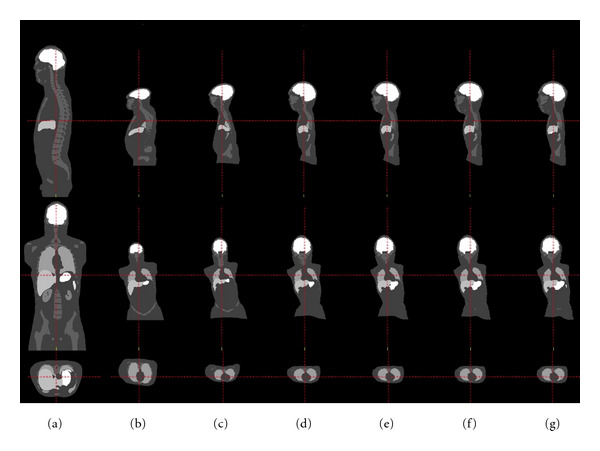
The robust sequence of transformations leading to the final mapping. Top row: sagittal slice, middle row: coronal slice, bottom row: axial slice. (a) Initial placement, (b) after affine registration, (c) after LDDMM landmark, and (d)–(g) after 1–4 iterations of MC-LDDMM.

The sequence of transformations used to generate the final mapping is illustrated in Figure 2. Each transformations for each pediatric patient were combined to yield a double precision displacement vector at each voxel of the adult template images. This transformation was trilinearly interpolated to map NURBS (nonuniform rational B-spline) surfaces defined in the XCAT phantoms to the coordinate system of the child.

### 3.2. Analysis of Computation

The bulk of the computational work was performed during cascading *α* MC-LDDMM, and as such, its performance was investigated more thoroughly. Four patients were selected, 2 males and 2 females, corresponding to the largest, smallest, and 1/3 interquartile sizes, denoted “small”, “med-small”, “med-large”, and “large”. Mappings were calculated on these patients using each of 1, 2, 4, 8, 16, and 24 (the maximum readily available) processors. The total computation time excluding input-output (IO) operations was analyzed for each case as well as the time spent in specific functions. This allowed us an understanding of how computation time scales with the number of processors used, and in particular identify at what point computation time begins to increase beyond what would be expected.

To be more thorough, the portions of the program that were affected by parallelization, including IO operations, were analyzed. Speedup, *c*_*n*_, due to parallelization on *n* processors was calculated (using “Amdahl's Law” [22] as in [23]) to be

(4) $$c_{n}=\frac{T\left(1\right)}{T\left(n\right)}=\frac{A+B}{A+B/n},$$

where *T*(*n*) is the total computation time for *n* processors, and for a single processor, *A* is the time spent that cannot be parallelized, and *B* is the time spent that can be parallelized. These two quantities are easily estimated from a two parameter fit to the above equation, which allows determination of the fraction of the total computational time that can be parallelized. Furthermore, the efficiency of parallelization was calculated according to

(5) $$e_{n}=\frac{c_{n}}{n}.$$

### 3.3. Accuracy of Mappings

Finally, the quality of the mappings produced was validated. For each segmented organ, a triangulated surface was produced using isosurface generation via marching tetrahedra [24]. For each template (target) vertex, the minimum distance to a vertex on the target (template) surface was measured. Distances for template and target vertices were combined, and their distributions were analyzed. Breaking down this analysis into categories allows an understanding of the robustness of the algorithm. As such, distributions were analyzed separately for males, females, as well as for each segmented organ.

## 4. Results

### 4.1. Computational Performance

A summary of the 4 subjects used to analyze computation performance is included in Table 2. The number of voxels in each image is shown in the second column, giving more precise meaning to the labels “small”, “med-small”, “med-large”, and “large”. The total number of iterations of gradient descent across the 4 applications of MC-LDDMM is shown in the third column. Due in part to adaptive stepsize selection in gradient descent, the number of iterations until convergence cannot be known before hand. In the fourth column, the product between number of voxels and number of iterations is shown as a rough approximation of the number of calculations used. This value can be used to better understand the timing results that follow. In particular the “med-small” case required the most iterations to converge, and the approximate number of calculations was much less for the “small” patient than for the other three. We stress that these four patients were chosen with interquartile spacing of their total number of voxels, as opposed to uniform spacing across number of voxels, or uniform spacing across number of calculations. Such a choice is reflective of the pediatric population to be examined, rather than properties of the algorithm itself.

The total computational time in hours, excluding IO operations, is shown in Table 3. The two largest components of calculations are also shown. Numerically integrating the velocity field using semi-Lagrangian interpolation is shown in Table 4, and trilinearly interpolating the images is shown in Table 5. Surprisingly, the longest amount of time was spent on the “med-small case”. While this is partially explained by the large number of iterations for this case shown in Table 2, other factors such as the specific implementation of the fast Fourier transform on a grid of this size, contribute as well.

**Figure 3:**
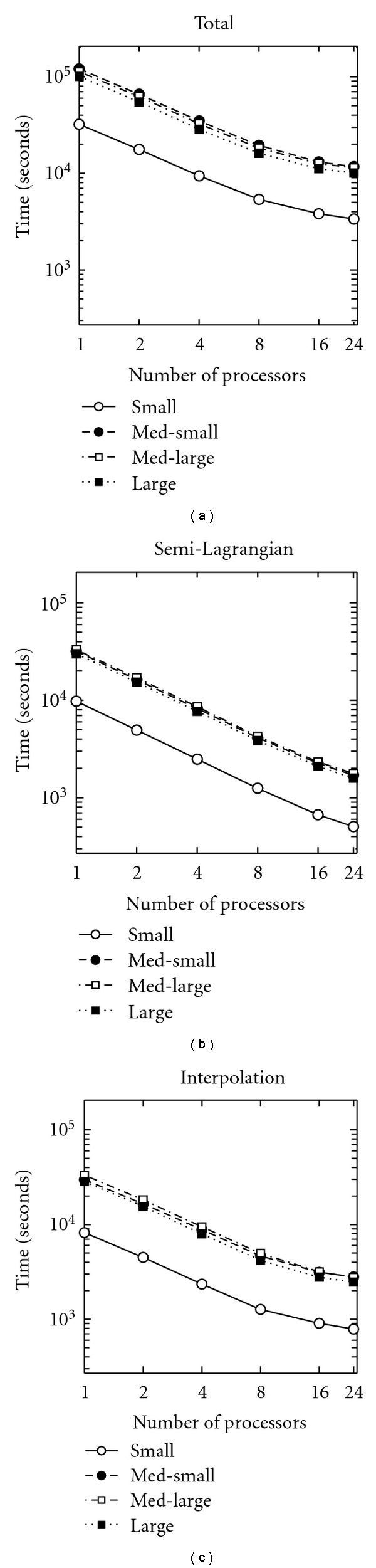
Time spent on computations for the four patients examined, plotted on a log-log axis. (a) Total time, (b) time in semi-Lagrangian advection, (c) time in image interpolation. Note that in (a) med-small takes the longest, followed by med-large, large, and small. In (b) and (c), the order of the first two is reversed.

To better understand this behavior, the same data is shown graphically, on a log-log axes in Figure 3.  Figure 3(a) shows the total time, Figure 3(b) shows the time spent calculating semi-Lagrangian advection, and Figure 3(c) shows the time spent interpolating images. It appears that computation time scales with number of processors up until around 8, when efficiency starts to break down.

**Figure 4:**
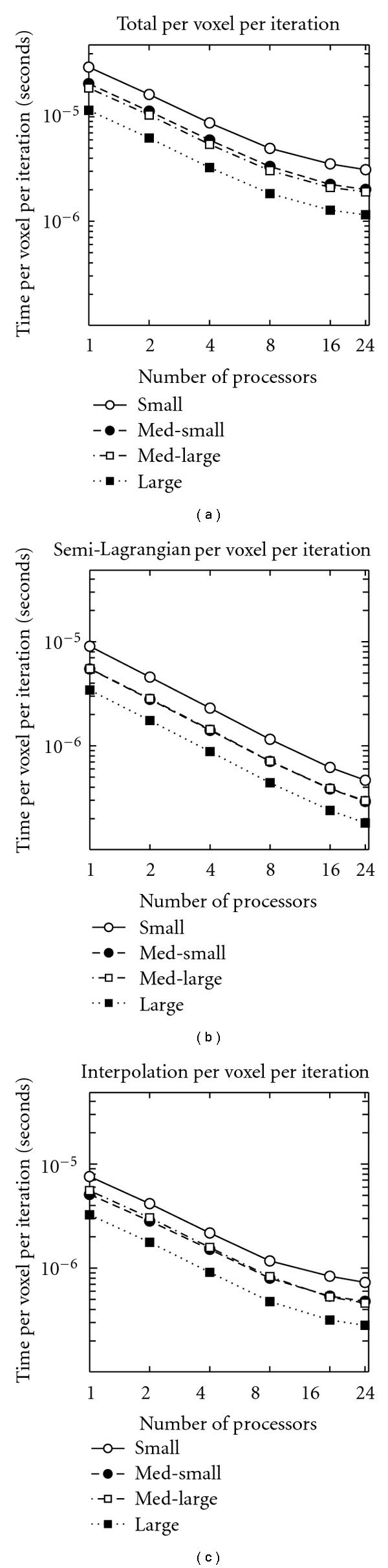
Time spent on computations, per image voxel per gradient descent iteration, for the four patients examined, plotted on a log-log axis. (a) Total time, (b) time in semi-Lagrangian advection, and (c) time in image interpolation. Note that in (a) small takes the longest, followed by med-small, med-large, and large. In (b) (for all processors) and (c) (from 1 to 8 processors), the order of the middle two is reversed.

Again, this data must be interpreted with caution, because the images used are different sizes and a different number of iterations of gradient descent is required to converge in each case, as shown in Table 2. Therefore, the timing data was also plotted after being normalized by total number of voxels times total number of iterations in Figure 4. It should be noted that the smallest image actually takes the most time per voxel per iteration, while the largest image takes the least.

**Figure 5:**
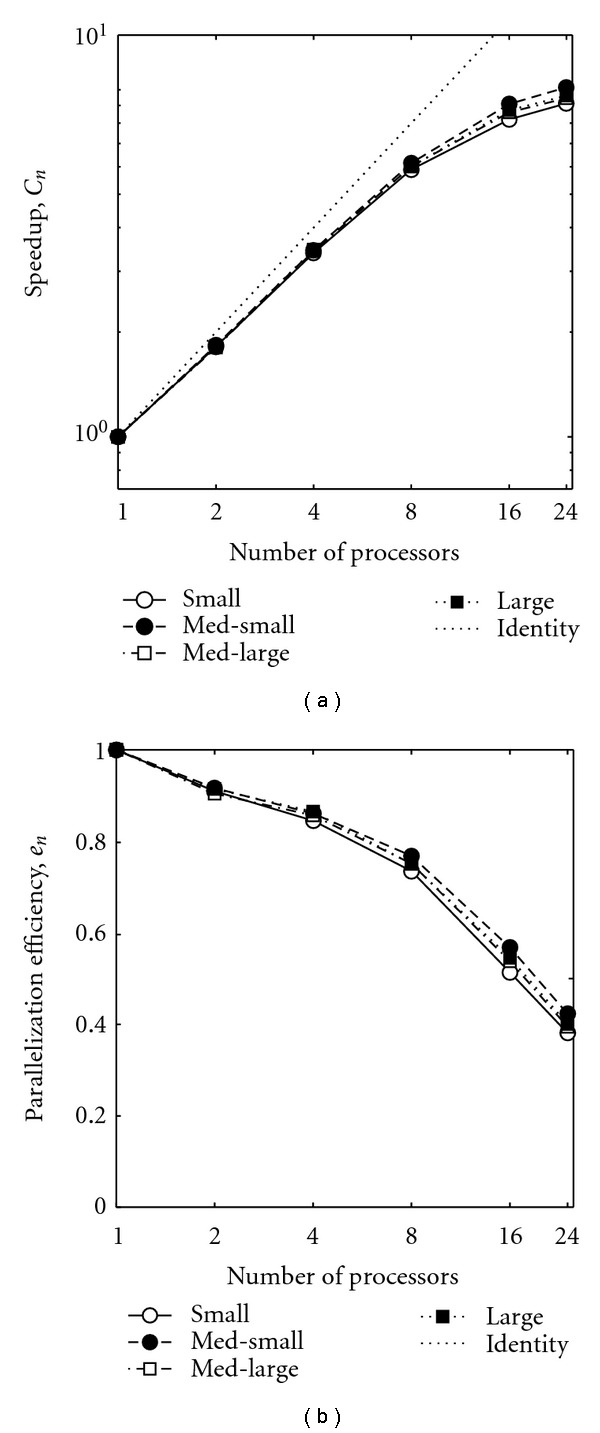
(a) Speedup due to parallelization (log scale) and (b) efficiency of parallelization (semilog scale), for the four patients examined. With the exception of “small” being uniformly the lowest, the order of the other varies as number of processors increases, and differences between each curve are quite small.

The speedup factor and efficiency were calculated according to (4) and (5) and are plotted in Figure 5. This analysis confirms and quantifies the sharp drop in efficiency beyond 8 processors. From a 2 parameter fit to the data in Figure 5(a), it was determined that 93.84% of the computation time is parallelized, demonstrating the effectiveness of our implementation.

### 4.2. Accuracy of Transformations

**Figure 6:**
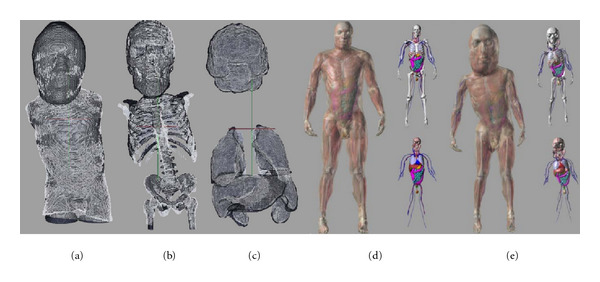
Triangulated surfaces from an example deformed adult template (white) and target child (black) are of (a) body, (b) bones, and (c) other organs. Adult male XCAT phantom is shown in (d), and an example custom dosimetry phantom is shown in (e).

To give a qualitative understanding of the mappings produced, an example of triangulated surfaces, for target and mapped template, are shown in Figure 6 with the body in Figure 6(a), the bones in Figure 6(b), and the other organs in Figure 6(c). One can see the quality of the mappings is good in most areas, the exceptions being the inferior-most regions, where the extent of template and target images vary, the scapula, where sliding motions between the nearby ribs and body surface are difficult to generate given the diffeomorphism constraint, and the sharp borders of some abdominal organs, whose curvature varies markedly from that of the template.

The mappings produced were used to generate customized dosimetry phantoms based on the adult XCATs. The adult male XCAT is shown in Figure 6(d) and an example pediatric dosimetry phantom is shown in Figure 6(e). Previous work has shown dosimetry measurements generated with these phantoms to agree within 10% percent of ground truth [5].

**Figure 7:**
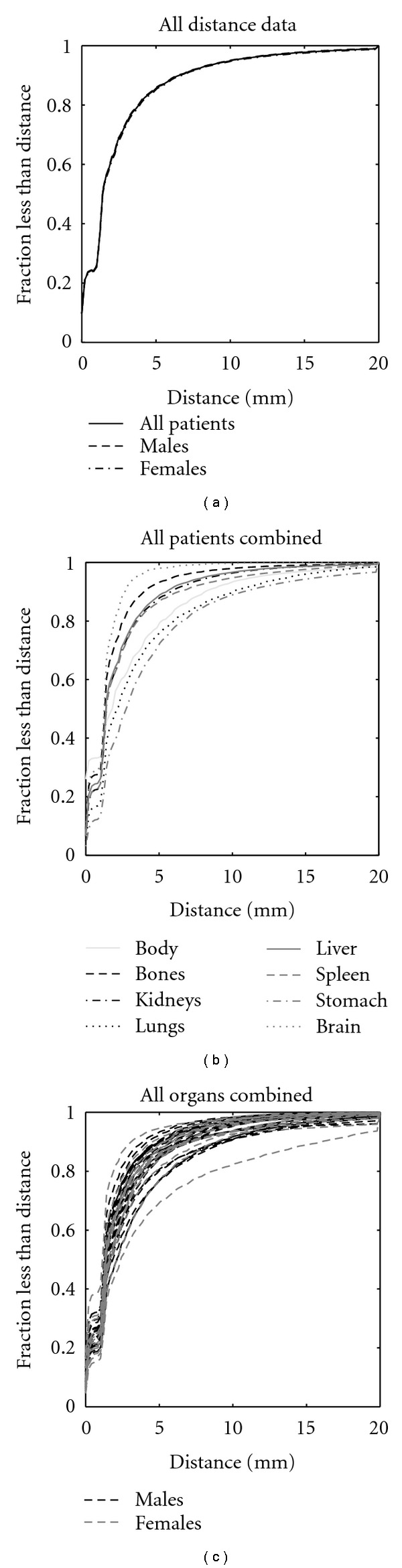
Cumulative distribution functions of final surface to surface distances are shown for all data and for all males and all females in (a), for individual organs with all patients combined in (b), and for individual patients with all organs combined in (c).

Cumulative distribution functions for final surface to surface distances are shown in Figure 7. They are shown for all patients pooled together as well as for males and females separately in Figure 7(a). The differences in accuracy, on average, between male and female patients is negligible. Additionally, distribution functions are shown for each organ in Figure 7(b). And they are shown for each of the 42 patients in Figure 7(c).

The results show that the majority of surfaces (a fraction of 1/*e* ~ 1 standard deviation of the vertices) agree within 2–4 mm or 1-2 voxels. Moreover, accuracy for females tends to be more variable than that for males, likely due to larger differences in body proportions between child and adult. Surprisingly, the least accurate case, apparent in Figure 7(c), is an average seeming patient of intermediate size between the med-small and med-large test cases. Furthermore, differences in accuracy for each organ are observed, where the brain is matched with the most fidelity and the stomach followed by lungs with the least fidelity. While these differences are small when compared to the voxel size, it is worth noting that the relatively poor performance for the stomach was likely due to its internal location and close proximity with many other abdominal structures, and the relatively poor performance of the lungs was likely due to large differences in curvature between the adult and child at the apexes and inferior borders.

## 5. Conclusions

This work presented an interesting application of diffeomorphic image registration, generating pediatric patient specific detailed dosimetry phantoms, made feasible on large scale due to parallel computing. The need for parallelization in deformable image registration is well recognized [23, 25, 26], and other authors have investigated parallelization of diffeomorphic registration from MASPAR [27] to GPU implementations [28].

The algorithm used here for generating full body maps involves a sequence of increasingly detailed transformations between adult templates and child images. This procedure ensures robustness to automate calculations across a wide range of pediatric patients but comes at the price high computational cost.

To overcome this cost, 93.84% of the algorithm computation time was parallelized. Running times for the various patients examined ranged from over 30 hours on a single processor to under 1 hour on 24 processors in parallel. An analysis of speedup and parallelization efficiency shows that performance begins to rapidly decline when implemented on more than 8 processors. As applications for LDDMM become more numerous and larger scale, an investigation of this issue will be necessary. It is likely that the effects of memory to cpu communication bandwidth, load balancing overhead (due to workload not evenly distributed across the available processors) play a major role.

The full body mapping algorithm is quite accurate for all the patients examined, with the majority of vertices defined on organ surfaces agreeing between template and target to within 2 voxels. Overcoming a main drawback of the diffeomorphism constraint, namely, forbidding sliding motions in the deformation, is the subject of ongoing research. One strategy we are currently investigating involves relabelling a strip of the segmented image, between two structures where sliding would be expected, as “background”. The XCAT phantoms generated are being further investigated for their accuracy and clinical utility.

While generating mappings using a sequence of transformations results in a robust algorithm for this application, it detracts from some of the theoretical appeal of LDDMM. Describing transformations by a single time-dependent vector field allows a rigorous study of anatomical variability. Future work will involve combining these transformations, for example, as described in [29], and beginning to engage in shape analysis of full bodies.

## Figures and Tables

**Table 1:** Segmented organs used for full body maps.

Organ	Weighting
Body	*σ*_1_ = 1
Bones	*σ*_2_ = 1
Kidneys	*σ*_3_ = 0.5
Lungs	*σ*_4_ = 1
Liver	*σ*_5_ = 1
Spleen	*σ*_6_ = 0.5
Stomach	*σ*_7_ = 0.5
Brain	*σ*_8_ = 1

**Table 2:** Summary of 4 subjects used to analyze computational performance.

Subject	Voxels	Iterations	*∼*No. of calculations
Small	2459200	439	1.08*e* + 09
Med-Small	6182224	942	5.82*e* + 09
Med-Large	9358976	640	5.99*e* + 09
Large	16082000	544	8.75*e* + 09

**Table 3:** Total timing (in hours) excluding IO operations.

Processors	Small	Med-small	Med-large	Large
1	8.94	33.5	31.3	28
2	4.9	18.2	17.3	15.2
4	2.62	9.68	9.05	7.92
8	1.49	5.41	5.07	4.47
16	1.06	3.64	3.5	3.1
24	0.935	3.25	3.17	2.8

**Table 4:** Semi-Lagrangian timing (in hours).

Processors	Small	Med-small	Med-large	Large
1	2.72	8.87	9.16	8.34
2	1.37	4.52	4.73	4.26
4	0.691	2.28	2.39	2.14
8	0.347	1.15	1.19	1.07
16	0.186	0.625	0.647	0.582
24	0.14	0.473	0.494	0.441

**Table 5:** Image interpolation timing (in hours).

Processors	Small	Med-small	Med-large	Large
1	2.28	8.27	9.24	7.91
2	1.25	4.59	5.07	4.3
4	0.653	2.45	2.63	2.21
8	0.352	1.29	1.38	1.16
16	0.251	0.869	0.88	0.771
24	0.219	0.776	0.767	0.685
